# Ultrahigh-resolution spectrometer based on 19 integrated gratings

**DOI:** 10.1038/s41598-019-46792-7

**Published:** 2019-07-15

**Authors:** An-Qing Jiang, Kai-Yan Zang, Hua-Tian Tu, Jian-Ke Chen, Wei-Jie Lu, Osamu Yoshie, Xiao-Ping Wang, Xiao-Dong Xiang, Young-Pak Lee, Bing Chen, Yu-Xiang Zheng, Song-You Wang, Hai-Bin Zhao, Yue-Mei Yang, Liang-Yao Chen

**Affiliations:** 1 Department of Optical Science and Engineering, Fudan University, Shanghai, China; 2 Graduate School of IPS, Waseda University, Fukuoka, Japan; 3 Department of Material Science and Engineering, SUSTC, Shenzhen, China; 4 Department of Physics, Hanyang University, Seoul, Korea; 5 Tucsen Photonics, Fujian, China

**Keywords:** Spectrophotometry, Imaging and sensing

## Abstract

Optical spectrometers play a key role in acquiring rich photonic information in both scientific research and a wide variety of applications. In this work, we present a new spectrometer with an ultrahigh resolution of better than 0.012 nm/pixel in the 170–600 nm spectral region using a grating-integrated module that consists of 19 subgratings without any moving parts. By using two-dimensional (2D) backsideilluminated complementary metal-oxide-semiconductor (BSI-CMOS) array detector technology with 2048 × 2048 pixels, a high data acquisition speed of approximately 25 spectra per second is achieved. The physical photon-sensing size of the detector along the one-dimensional wavelength direction is enhanced by a factor of 19 to approximately 428 mm, or 38912 pixels, to satisfy the requirement of seamless connection between two neighboring subspectral regions without any missing wavelengths throughout the entire spectral region. As tested with a mercury lamp, the system has advanced performance capabilities characterized by the highest k parameter reported to date, being approximately 3.58 × 10^4^, where k = (working wavelength region)/(pixel resolution). Data calibration and analysis as well as a method of reducing background noise more efficiently are also discussed. The results presented in this work will stimulate further research on precision spectrometers based on advanced BSI-CMOS array detectors in the future.

## Introduction

Optical spectrometers play a significant role in profoundly exploring the nature of matter by extracting rich optical information with high precision in modern scientific research and a broad variety of applications. These instruments are typically used in spectroscopic analysis to identify materials^1–5^. They can also be integrated into other optical instruments to acquire useful spectral information in a wide variety of fields^6,7^. Continuous development efforts are being made to improve the precision and portability of spectrometers in many different configuations^8–14^.

The modern broadband echelle spectrometer possesses the unique feature of an ultrahigh spectral resolution based on a special system design consisting of a grating with a very low groove density, which allows it to work in a region spanning more than 90 spectral orders^15^, resulting in nonuniform resolution and lower or even zero efficiency in some bands where no spectral signal can be detected. These zero-efficiency bands will constitute as much as approximately 10% of the entire spectral region due to system limitations^9^.

Grating-based spectrometers with various optical and mechanical configurations are still widely used to pursue the goals of high diffraction efficiency and resolution. In traditional grating-based spectrometer designs, however, a mechanical motion process with fine grating scanning steps is required to obtain full coverage of the spectral lines in the desired wavelength range. Such mechanical-motion-based operation will not only limit the data acquisition speed but also reduce the measurement reliability of the spectrometer system over the long term.

With the use of a 2D array of advanced CMOS and charge-coupled device (CCD) detectors, a single grating-integrated module consisting of multiple subgratings can be constructed to achieve a spectrometer with a high and relatively uniform diffraction efficiency over a broad spectral range. Multifold spectra can be simultaneously imaged on the focal plane of the detector by individual subgratings with high diffraction efficiency (on the order of β = 1), which can be optimally chosen and spectrally arranged at the blazed wavelength λ_b_ in accordance with the specific system design in parallel data acquisition mode^14,16,17^. Thus, such a grating-integrated spectrometer can achieve superior performance compared with a traditional spectrometer, with 3 key advantages: (1) a wide range of measurable wavelengths with the same focal length, (2) an increase in the spectral resolution without any significant change in the overall system size, and (3) a great increase in the data acquisition speed, improving the long-term system reliability in applications without any moving elements.

A spectrometer with ultrahigh resolution is required to identify elements based on their unique spectral “fingerprints^1^”. In particular, for many metals, these “fingerprints” lie in the 170–600 nm spectral region. In this work, therefore, we propose a new spectrometer with an ultrahigh resolution of better than 0.012 nm/pixel in the 170–600 nm spectral region using a grating-integrated module that consists of 19 subgratings without any moving parts. A high data acquisition speed of approximately 25 spectra per second is achieved by using a 2D BSI-CMOS array detector with a 2048 × 2048 pixel density. As tested with a mercury lamp, the system demonstrates high performance characterized by the highest value of the k parameter reported to date, being approximately 3.58 × 10^4^, where k = (working wavelength region)/(pixel resolution). Data analysis, including a method of more effectively reducing background noise, is also discussed in detail. The results presented in this work will stimulate further research on high-precision spectrometers based on advanced 2D BSI-CMOS array detectors in the future.

## Experimental Configuration

**Figure 1:**
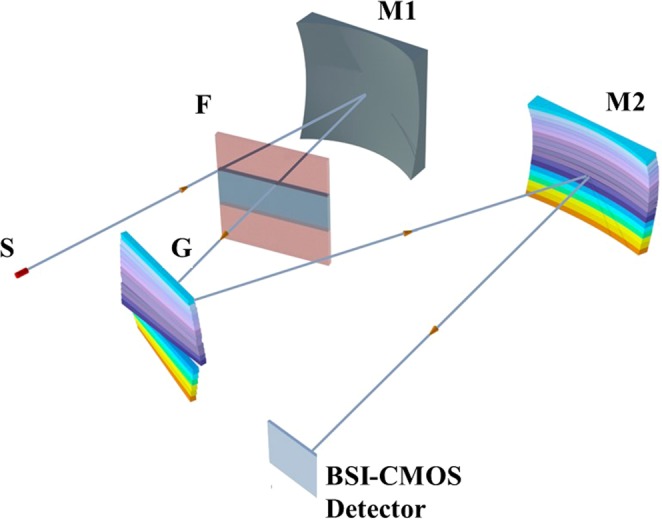
Schematic illustration of the spectrometer system. S is an optical slit, and G is the grating-integrated module consisting of 19 subgratings. The light is parallelized by the spherical mirror M1. The grating-diffracted light is then focused by the toroidal mirror M2 onto the focal plane of the 2D BSI-CMOS array detector. F is the filter set used to effectively eliminate the high-order (β ≥ 2) components of the diffracted light corresponding to each subspectral region, where the gray-colored region is blank (without filters).

A schematic illustrating the configuration of the developed Czerny-Turner-type spectrometer with a high-density grating-integrated module is shown in Fig. 1. The grating module G consists of 19 subgratings arranged in parallel along the direction perpendicular to the light incidence plane. By means of fiber coupling, the light signal is input through the slit S, which has a width of 10 µm and a height of 0.8 mm. The light reflected by the spherical mirror M1, which has a focal length of 500 mm, will become parallel and enter the grating module. The first order (β = 1) of the grating diffraction with higher efficiency is used. The filter set F is used to effectively eliminate the high-order (β ≥ 2) components of the diffracted light corresponding to each subspectral region. The toroidal mirror M2 has two focal lengths of 500 mm and 604 mm along the directions parallel and perpendicular, respectively, to the light incidence plane; it is used to focus the diffracted light onto the focal plane of the 2D BSI-CMOS array detector (Dhyana 90 UV camera), which has dimensions of 2048 × 2048 pixels with a pixel size of 11 × 11 µm^2^ and a MgF_2_ transparent window, allowing it to operate in the 170–1000 nm wavelength region with a peak efficiency of approximately 90% at 400 nm.^18^. This makes the integrated spectral image of 19 gratings have the size ≤ 22.5 mm along the directions perpendicular to the light incidence plane at the focal position of 500 mm, and be fully filled into the focal plane of the CMOS detector. Each subspectral region, each with dimensions of 108 × 2048 pixels, thus has a photon-sensing size of approximately 1.2 mm × 22.5 mm along the directions perpendicular and parallel, respectively, to the light incidence plane on the focal plane of the array detector.

The spectral difference measured between two wavelengths corresponding to two neighboring pixels can be resolved with a maximum resolution of

1 $${\rm{\Delta }}{\lambda }_{\max }=\frac{dw}{f} > \frac{dw\,\cos \,\theta }{f}$$

where *θ*, *f* and *d* are the diffraction angle, the focal length of the mirror and the groove width of the grating, respectively, and *w* is the width of a single pixel, which is equal to 11 µm and matches the slit width of 10 µm. When a grating with a groove density of 1800 lines/mm is used in the grating-integrated module, d ≈ 556 nm in this design. The calculated Δλ_max_ is approximately 0.012 nm, implying that the spectrometer has a theoretical resolution of better than 0.012 nm/pixel. According to the theoretical expectations regarding the operation of the spectrometer in the 170–600 nm wavelength region, therefore, a high spectral performance characterized by a high k factor can be realized for the spectrometer; specifically, k = (working wavelength region)/(pixel resolution) = 35833 without any moving elements.

**Figure 2:**
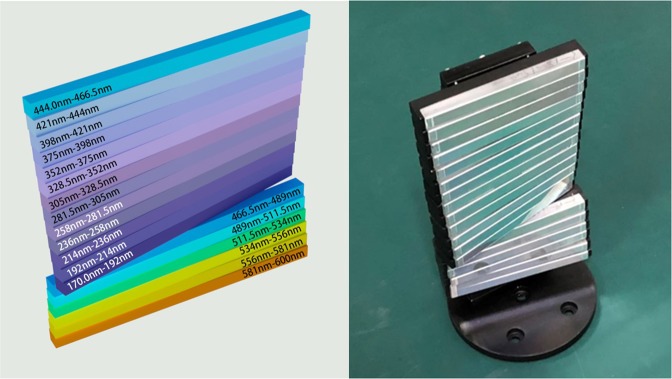
The high-density grating-integrated module, consisting of 19 subgratings, one corresponding to each individual subwavelength region.

Usually, the light intensity is not uniformly distributed in the exit core angle region of the fiber; instead, the light intensity close to the beam center will be higher than that in the edge region. Moreover, the efficiency of measuring the spectral signal in the ultraviolet region will be lower than that in the visible region due to many factors. For an optimal system design, therefore, the subgratings in the module are assembled with a certain flexibility, and those that work in shorter-wavelength regions are placed closer to the beam center, as shown in Fig. 2, such that the gratings correspond to the following individual spectral regions: (1) 444–466.5 nm, (2) 421–444 nm, (3) 398–421 nm, (4) 375–398 nm, (5) 352–375 nm, (6) 328.5–352 nm, (7) 305–328.5 nm, (8) 281.5–305 nm, (9) 258–281.5 nm, (10) 236–258 nm, (11) 214–236 nm, (12) 192–214 nm, (13) 170–192 nm, (14) 466.5–489 nm, (15) 489–511.5 nm, (16) 511.5–534 nm, (17) 534–556 nm, (18) 556–581 nm, and (19) 581–600 nm. Each subgrating has dimensions of 70 mm (length) and 5.7 mm (height) along the directions parallel and perpendicular, respectively, to the light incidence plane.

The fixed angle *θ*_λ_ of each grating is calculated according to Equation (2) with respect to the horizontal direction parallel to the focal plane of the CMOS detector, where the fixed system constant α is equal to 9 degrees (half of the angle between the incident and diffracted beams onto and from the grating, respectively) in the design^14,16,17^. Regarding to the subgrating arrangement as shown in Fig. 2, the values of the central wavelength *λ*_0_ and fixed angel *θ*_λ0_ for each subgrating are that: (1) 455.25 nm (24.509 deg.), (2) 432.5 nm (23.21 deg.), (3) 409.5 nm (21.9 deg.), (4) 386.5 nm (20.261 deg.), (5) 363.5 nm (19.343 deg.), (6) 340.25 nm (18.062 deg.), (7) 316.75 nm (16.776 deg.), (8) 293.25 nm (15.449 deg.), (9) 269.75 nm (14.229 deg.), (10) 247 nm (13.007 deg.), (11) 225 nm (11.831 deg.), (12) 203 nm (10.66 deg.), (13) 181 nm (9.493 deg.), (14) 477.75 nm (25.807 deg.), (15) 500.25 nm (27.119 deg.), (16) 522.75 nm, (28.447 deg.), (17) 545 nm (29.776 deg.), (18) 568.5 nm (31.2 deg.), (19) 590.5 nm (32.553 deg.).

2 $${\theta }_{\lambda }={\sin }^{-1}(\frac{\lambda }{2d\,\cos \,\alpha })$$

The blazed wavelengths are chosen to ensure higher diffraction efficiencies at 250 nm and 500 nm for the gratings operating in the 170–398 nm and 398–600 nm spectral regions, respectively. For the filter set F, as shown in Fig. 1, there is a blank window without any filter in the 170–328.5 nm spectral region, and there are two filters with a cut-off wavelength of 320 nm to effectively block the high-order (β ≥ 2) components of the diffracted light in the 328.5–600 nm spectral region.

The background noise arising from various mechanisms depending on factors, such as temperature, stray light, working time, and nonuniform pixel properties of the detectors is an issue to be solved in the data reduction procedure. During analysis, an image of the background intensity (I_b-image_) without any signal is initially measured for all 2048 × 2048 pixels of the detector under the required experimental conditions, and a raw image of the signal intensity (I_r-image_) is then measured under the same experimental conditions. For further analysis of the image data, finally, a true, clean spectral image of the signal intensity (I_s-image_) can be obtained by subtracting I_b-image_ from I_r-image_ for all pixels of the detector as follows:

3 $${{\rm{I}}}_{{\rm{s}}-{\rm{image}}}={{\rm{I}}}_{{\rm{r}}-{\rm{image}}}-{{\rm{I}}}_{{\rm{b}}-{\rm{image}}}$$

Due to imperfections in the optical elements and misalignment of the spectrometer, aberration error affects the spectral resolution, and it is usually difficult to completely eliminate such error during system design and construction^19–21^. In our experimental system, there are approximately 108 wavelength lines corresponding to the 108 × 2048 pixels in each subspectral region. In the calibration procedure, two steps are performed to overcome the aberration error, as follows. In the first step, a well-calibrated high-precision monochromatic light source is used to determine the relationship between the pixel position x(λ_n_) and the wavelength λ_n_ for each pixel n along the spectral direction. This relationship can be well fitted by a binomial function:

4 $${x}_{m}({\lambda }_{n})={a}_{n}+{b}_{n}\cdot {\lambda }_{n}+{c}_{n}\cdot {\lambda }_{n}^{2},$$

where m represents the m-th pixel line along the direction perpendicular to the light incidence plane in the subspectral region. The constants a_n_, b_n_ and c_n_, which characterize the dispersion relationship between the pixel position and wavelength, can be precisely determined via the calibration procedure. In the second step, the spectral intensities I_m_(λ_n_) corresponding to the same wavelength λ_n_ at pixel line m and position n can be analyzed to obtain the average intensity I(λ_n_) of M lines (M ≤ 108) in a given subspectral region as follows: I(λ_n_) = [Σ_m_I_m_(λ_n_)]/M. By means of this binomial calibration procedure based on the 2D pixel-to-wavelength analysis method, both the signal-to-noise ratio and the spectral resolution of the spectrometer can be significantly improved.

All optical and mechanical parts, including the module containing each fixed subangle to integrate and mount 19 subgratings as shown in Fig. 2, in the system were designed using computer-aided design (CAD) software and were produced via high-precision computer-assisted machining. After assembly and alignment, all parts, including the subgratings, were fixed with optical glue to ensure the long-term stability of the system.

## Results and Discussion

**Figure 3:**
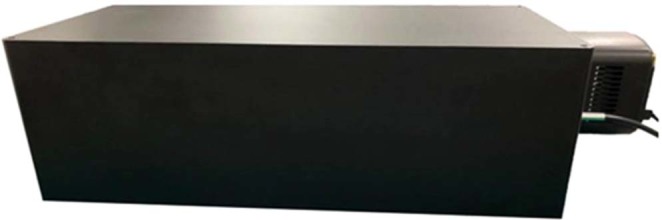
A photograph of the prototype solid spectrometer constructed in accordance with the proposed design with dimensions of 570 (length)x270 (width)x180 (height) mm, not including the BSI-CMOS camera.

A prototype solid spectrometer with a grating module consisting of 19 subgratings was successfully constructed in accordance with the proposed design, as shown in Fig. 3. The dimensions are 570 (length)x270 (width)x180 (height) mm, not including the BSI-CMOS camera. Software for data acquisition and analysis was written and compiled for both the calibration and measurement procedures.

**Figure 4:**
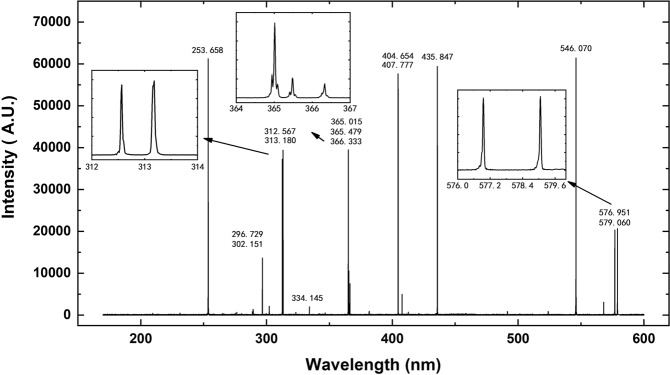
Spectral lines of a Hg lamp measured in the 170–600 nm wavelength region, with zoomed-in views of the well-resolved triple and twin spectral lines of elemental Hg shown in the insets.

A Hg lamp with spectral “fingerprints” in the 170–600 nm wavelength region was used to test the performance of the spectrometer. The results are shown in Fig. 4, where the insets show zoomed-in views of typical triple and twin spectral lines of elemental Hg. These spectral lines originate from stimulated emissions from the Hg atom and are associated with the transitions of electrons between its internal energy states. For example, the triple spectral lines of Hg located at 365.015 nm, 365.484 nm and 366.328 nm correspond to the transitions from the 6d^3^D_3_, 6d^3^D_2_ and 6d^1^D_2_ states, respectively, to the 6p^3^P_2_ state. The twin-line structure located at 576.961 nm and 579.07 nm corresponds to the transitions from the 6d^3^D_2_ and 6d^1^D_2_ states to the 6p^1^P_1_ state^22^. The characteristic spectral lines of the Hg atom can be used to evaluate the resolution of the spectrometer. It is clear from Fig. 4 that these triple and twin spectral lines as well as other atomic lines can be clearly resolved without any ambiguity and are in good agreement with the expected locations of the Hg lines. It can also be seen that the spectral line located at 253.658 nm still has a relatively high intensity due to the optimal grating arrangement in the ultraviolet region, as mentioned above, by virtue of the special design considerations.

**Figure 5:**
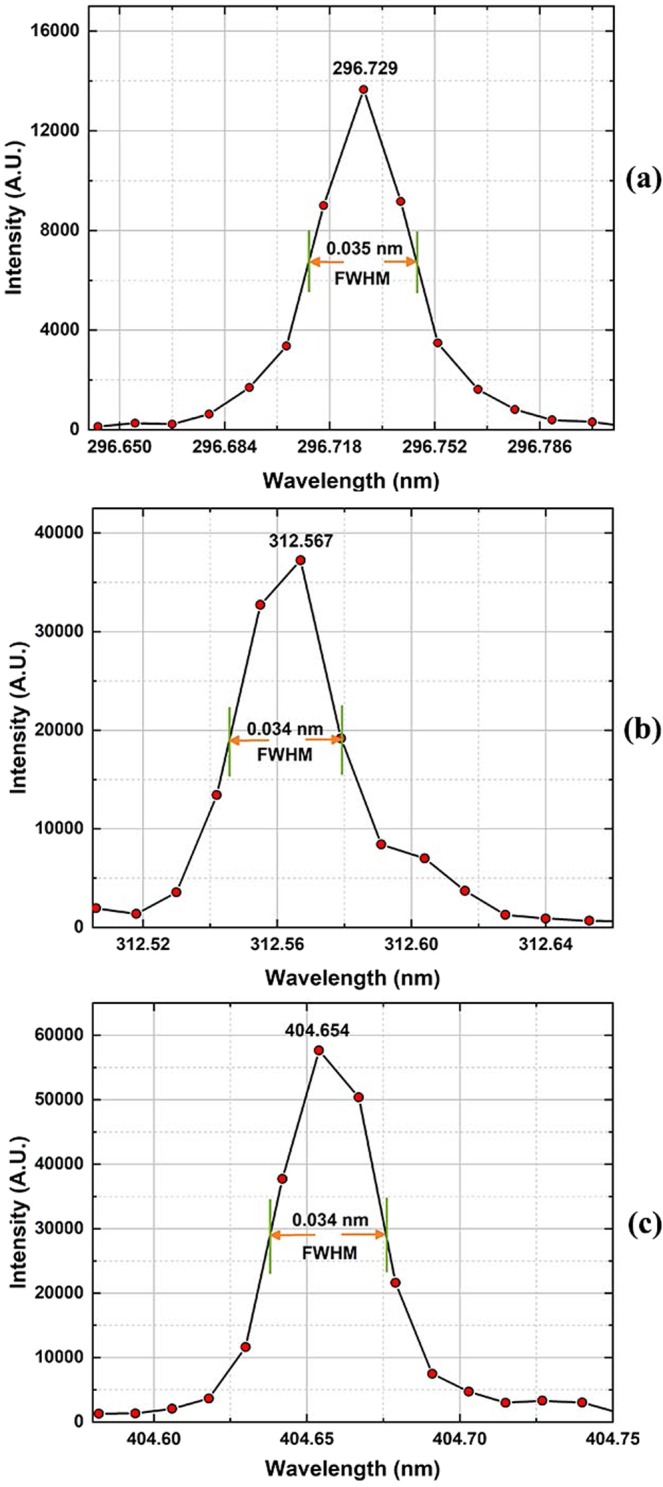
The fine spectral lines of elemental Hg located at 296.728 nm, 312.567 nm and 404.654 nm were used to evaluate the spectral resolution, demonstrating that the pixel resolution (∆λ(FWHM)/3 = δλ ≤ Δλ_max_) is better than 0.012 nm/pixel, consistent with the design expectations.

The measured spectral resolution ∆*λ* is defined by the full width at half maximum (FWHM) of a spectral line and is not equal to the pixel resolution presented in Equation (1). The different structures of Hg atoms belonging to different isotopes will give rise to some weak satellite lines in the spectrum. To avoid the resultant satellite line-broadening effect, which will induce measurement error, the relatively fine and clean spectral lines located at 296.279 nm, 312.567 nm and 404.654 nm, as shown in Fig. 4, were used to evaluate the spectral resolution, with the results shown in Fig. 5. The FWHM of a spectral line should span at least 3 pixels, which are equivalent to 3 fixed beam-exiting slits. Based on the data analysis, the measured ∆λ(FWHM) values of the spectral lines at 296.279 nm, 312.567 nm and 404.654 nm were found to be in the range of approximately 0.034–0.035 nm, thus confirming a pixel resolution (∆λ(FWHM)/3 = δλ ≤ Δλ_max_) of better than 0.012 nm/pixel, consistent with the design expectations.

Since this spectrometer does not require a wavelength-scanning step, which might adversely affect the measurement accuracy due to mechanical errors after long-term operation, the ultrahigh-resolution spectrometer achieved in this work demonstrates the advantages of a broad working wavelength range, a high spectral resolution and a fast data acquisition speed during practical operation without any moving parts. Through the use of a 2D array detector with a grating-integrated module consisting of 19 subgratings, the actual physical photon-sensing size along the one-dimensional wavelength direction is enhanced by a factor of 19 to approximately 428 mm, or 38912 pixels, providing enough pixel capacity to ensure a seamless connection between two neighboring subspectral regions without any missing wavelengths, which usually arise in the design of an echelle spectrometer^15^.

The working spectral region, Δλ(working region), is equal to 430 nm, and the pixel resolution δλ in this region is better than 0.012 nm/pixel, in accordance with the system design. The value of the k parameter (Δλ/δλ) that characterizes the performance of the spectrometer is thus approximately 3.58 × 10^4^, which is the highest value reported to date. By using an advanced scientific-grade 2D BSI-CMOS array detector with a relatively large photon-sensing size, we can take full advantage of the uniformity of the opto-electronic properties of the detector on the imaging plane, with high quantum efficiency and extremely low noise at a given constant temperature, which can be precisely and uniformly set and controlled down to −10 °C during operation. The high-speed frame-scan rate of the BSI-CMOS array detector can also assist in applying the practical approach of capturing multiple spectral images for use in data acquisition and reduction to effectively enhance the signal-to-noise ratio.

## Conclusion

In this study, by using a state-of-the-art 2D BSI-CMOS array detector combined with a grating-integrated module consisting of 19 gratings, a spectrometer with an ultrahigh spectral resolution of better than 0.012 nm/pixel in the 170–600 nm wavelength region was constructed. The physical photon-sensing size of the detector along the one-dimensional wavelength direction is enhanced by a factor of 19 to approximately 428 mm, or 38912 pixels, thereby satisfying the requirement of seamless connection between two neighboring subspectral regions without any missing wavelengths throughout the entire spectral region. Methods of 2D calibration and noise reduction during data analysis have been discussed and applied to effectively improve the spectral resolution and signal-to-noise ratio. The spectrometer has a data acquisition speed of approximately 25 spectra per second without any mechanical moving parts. As tested with a mercury lamp, the system has advanced performance capabilities characterized by the highest value of the k parameter reported to date, being approximately 3.58 × 10^4^, where k = (working wavelength region)/(pixel resolution). The results presented in this work will stimulate further research on precision spectrometers based on advanced 2D BSI-CMOS array detectors with high-density grating-integrated modules in the future.
